# COVID-19 mortality among pregnant women in Mexico: A retrospective cohort study

**DOI:** 10.7189/jogh.10.020512

**Published:** 2020-12

**Authors:** Mónica Ríos-Silva, Efrén Murillo-Zamora, Oliver Mendoza-Cano, Xóchitl Trujillo, Miguel Huerta

**Affiliations:** 1 Centro Universitario de Investigaciones Biomédicas, Universidad de Colima, Colima, Mexico; 2 Cátedras CONACyT, Universidad de Colima - Centro Universitario de Investigaciones Biomédicas, Colima, México; 3 Departamento de Epidemiología, Unidad de Medicina Familiar No. 19, Instituto Mexicano del Seguro Social, Colima, Colima, México; 4 Facultad de Ingeniería Civil, Universidad de Colima, Coquimatlán, Colima, México

## Abstract

**Background:**

Pregnant women have been classified as at risk for COVID-19 due to previous experience with influenza and other coronaviruses. The objective of this study was to identify risk factors for the complications and death in women of childbearing age and pregnant women with suspected COVID-19.

**Methods:**

This retrospective cohort study was conducted from the beginning of the epidemic in Mexico until May 25, 2020. All women of childbearing age (13-49 years) from the open national COVID-19 database from the Ministry of Health of Mexico were considered for eligibility. SARS-COV-2 infection was confirmed or ruled out by RT-qPCR. We performed a bivariate and multivariable analysis to estimate mortality risk.

**Results:**

Ten (2.2%) pregnant women with confirmed COVID-19 died. Positive pregnant patients did not have a higher risk of complications (admission to the ICU, pneumonia, or requirement for mechanical ventilation) or death than the controls. In the multivariate analysis, only history of diabetes and chronic kidney disease remained independently associated with death in the positive cohort. Seven (0.6%) pregnant women with a negative test died. In bivariate analysis, pregnant patients with a positive test had a higher risk of death than pregnant patients with a negative test (relative risk (RR) = 3.87, 95% confidence interval (CI) = 1.48-10.12), but no higher risk was found than in non-pregnant women with a positive test (RR = 0.82, 95% CI = 0.44-1.53), and 60-day mortality did not significantly differ among pregnant patients with or without a positive test (hazard ratio (HR) = 0.40, 95% CI = 0.12-1.30) or between COVID-19-positive patients who were pregnant or not pregnant (HR = 0.74, 95% CI = 0.35-1.56).

**Conclusions:**

Pregnant patients do not have a greater risk of complications or death from COVID-19 than non-pregnant patients. The presence of diabetes mellitus and chronic disease increases the risk of death in women of childbearing age, but not specifically in pregnant patients.

The COVID-19 pandemic caused by SARS-CoV-2 has generated worldwide alarm and mobilized all health services. Sectors of the population that are particularly vulnerable to serious disease and increased mortality have been identified, including patients with chronic degenerative diseases [1]. In addition, pregnant patients have been classified as high risk due to previous experience in pandemic influenza [2] and other diseases caused by other coronaviruses [3], but epidemiological studies have not conclusively demonstrated increased mortality in pregnant women compared to non-pregnant women [4]. To date, no specific authorized treatment is available for COVID-19, and some promising drugs do not have enough information regarding their safety during pregnancy. Furthermore, some therapeutic supportive measures, such as the prone position, are not possible in the last trimesters of pregnancy [5,6].

In Mexico, the general maternal morbidity and mortality has been estimated as a Maternal Mortality Ratio (MMR) of 34.5 deaths for every 100 000 estimated births [7]. Risk factors, such as teenage pregnancies, diabetes, obesity, and hypertension, have a higher prevalence [8,9]. Mexican health authorities have reported less use of health reproductive services by women of childbearing age, suggesting that the presence of SARS-CoV-2 infection could increase maternal morbidity and mortality rates. This increase in maternal mortality due to indirect factors of the use and availability of health services is estimated to range from 8.3 to 38.6% per month in low and middle income countries[10]. Given that COVID-19 is an emerging disease, information on its epidemiology in pregnant women is limited. Thus, the objective of the present study was to identify risk factors for the presence of complications and death in women of childbearing age and pregnant women with suspected COVID-19.

## METHODS

### Study design and participants

This retrospective cohort study included women of childbearing age (13 to 49 years)[11] from the open national database of COVID-19 [12] from the Ministry of Health of Mexico. All women of childbearing age included from the beginning of the epidemic in Mexico until May 25, 2020, were considered for eligibility.

### Procedures

Data were extracted from the open database, which includes anonymous information on cases studied by the General Directorate of Epidemiology of the Ministry of Health for epidemiological surveillance purposes; it includes suspected cases, negative cases, and confirmed cases. Suspicious cases were cataloged according to the operational definition issued by the Ministry of Health of Mexico, which includes the presence in the last 7 days of at least two of three symptoms, cough, fever, or headache, in addition to at least one of the following symptoms: dyspnea, arthralgia, myalgia, sore throat, runny nose, conjunctivitis, or chest pain. SARS-CoV-2 infection was confirmed or ruled out by laboratory tests utilizing quantitative reverse transcription polymerase chain reaction (RT-qPCR). The epidemiological surveillance network established that the tests would be carried out in 10% of the suspected cases cataloged as mild and 100% of severe cases (ie, anyone who meets the definition of a suspected case and also has difficulty breathing and is hospitalized). The database includes all cases in which a blood test was performed. The variables included in this database were sex; age; whether the patient received outpatient care or required hospitalization; presence of pregnancy; smoking history; date of onset of symptoms, admission to hospital, and death if applicable; history of diabetes mellitus (DM), chronic obstructive pulmonary disease (COPD), asthma, immunosuppression, hypertension, cardiovascular disease, obesity, chronic kidney disease, or diagnosis of other unspecified comorbidity; presence of complications, such as diagnosis of pneumonia, mechanical ventilation required, and intensive care in intensive care unit (ICU). The database also reports demographic variables corresponding to the geographic distribution of the cases. Women with missing clinical or epidemiological data were excluded. The database is available under an international license, CC-BY-ND 4.0.

The main outcome was to establish whether pregnant women with laboratory-confirmed COVID-19 have a higher risk of mortality vs non-pregnant women of childbearing age with laboratory-confirmed COVID-19 and pregnant women with suspected COVID-19 but with a negative test. The secondary outcomes were to establish whether the presence of chronic degenerative diseases increases the mortality rate in pregnant women with laboratory-confirmed COVID-19.

### Statistical analysis

We performed a descriptive analysis of the data; the continuous variables were expressed as medians and interquartile ranges (IQRs) and the categorical variables as frequencies and proportions (%). First, statistical analyses were performed in pregnant women with SARS-COV-2 positive tests using non-pregnant women of childbearing age as the control group. Next, statistical analyses were performed in pregnant women with suspected COVID-19 using pregnant women with a negative SARS-COV-2 test as the control group. In the bivariate analysis, a χ^2^ test was used to establish the association between the presence of pregnancy or a positive test and the presence of comorbidities reported in the database (DM, COPD, asthma, immunosuppression, hypertension, obesity, cardiovascular disease, chronic kidney disease, smoking, and other comorbidity), as well as the association of pregnancy with the presence of complications (pneumonia, admission to ICU, need for mechanical ventilation, and/or death), the association between the presence of comorbidities and the type of care required by the patient (outpatient vs hospitalization), and the association of comorbidities and death. We estimated the relative risk (RR) with 95% confidence intervals (CIs). For the quantitative variables, the data distribution was estimated using the Kolmogorov-Smirnov test. For a non-parametric distribution we used the Mann-Whitney U test. For the multivariate analysis, Cox proportional risk models for the risk of 60-day COVD-19 mortality in women of childbearing age considering the time from the onset of symptoms included all comorbidities adjusted for age and pregnancy status. Cox proportional risk models were also used to establish the risk of a positive test in pregnant women considering the time form the onset of symptoms until health service contact. We also estimated average hazard ratios using Mantel-Haenszel estimators. All statistical tests were two-tailed, and significance was set at α = 0.05. Statistical analysis was performed with SPSS version 20.0 (IBM corporation,Armonk NY, USA), for the realization of the graphs was used GraphPad Prism 6.01(GraphPad Software, San Diego CA, USA).

## RESULTS

**Figure 1:**
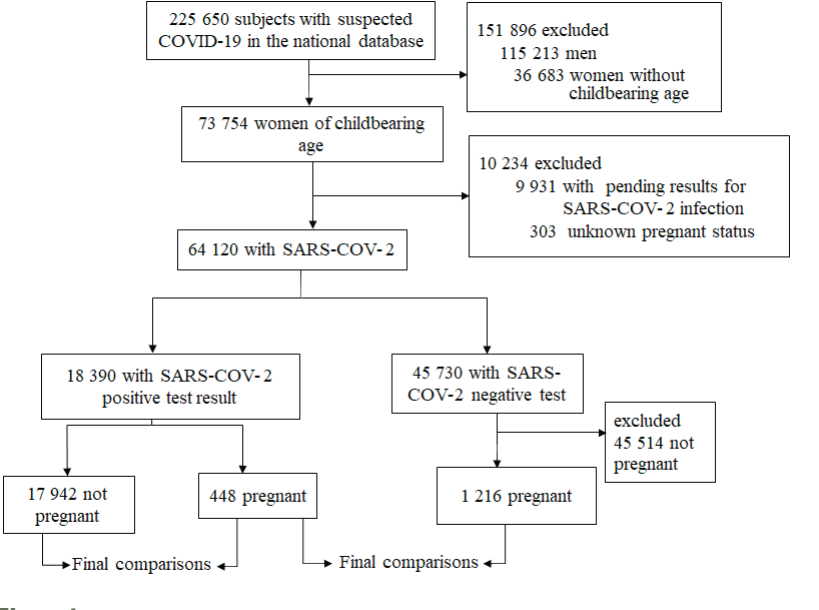
Flowchart of the study.

Between February 28, 2020 (the first confirmed case in Mexico) and May 25, 2020, a total of 225 650 suspected cases of COVID-19 were identified, 48% corresponding to women. The median age of women with a positive test was 36 years (IQR = 29-43), and 448 (2.49%) of these women were pregnant (Figure 1). The cohort included 19 636 women; 1486 (7.5%) had DM, 83 (0.4%) COPD, 853 (4.3%) asthma, 259 (1.32%) immunosuppression, 1710 (8.3%) hypertension, 3724 (18.9%) obesity, 209 (1.06%) cardiovascular disease, 209 (1.06%) chronic kidney disease, 1093 (5.5%) smoking, and 773 (3.9%) other not specified comorbidity.

### Women with positive SARS-COV-2 test

The median age of pregnant women was significantly lower than that of non-pregnant controls. The most frequent chronic condition in both pregnant and non-pregnant women was obesity, but pregnant patients had a higher risk of having another not specified comorbidity (RR = 1.98, 95% CI = 1.39-2.81; **Table 1**).

**Table 1:** Clinical characteristics of women of childbearing age with COVID-19

	No. pregnant positive tests (%) (n = 448)	No. not pregnant positive tests (%) (n = 17 942)	*P* value *	No. pregnant negative tests (%) (n = 1216)	*P* value†
Age (median, IQR)	29 (25-33)	37 (30-43)	<0.001	28 (23-32)	<0.001
DM	17 (3.7%)	1440 (8.2%)	0.001	29 (2.4%)	0.1
COPD	1 (0.2%)	79 (0.4%)	0.4	3(0.2%)	0.4
Asthma	11 (2.4%)	783 (4.3%)	0.04	59 (4.9%)	0.03
Immunosuppression	7 (1.5%)	228 (1.2%)	0.5	24 (2.0%)	0.5
Hypertension	17 (3.7%)	1655 (9.2%)	<0.001	38 (3.1%)	0.5
Obesity	59 (13.1%)	3563 (19.9%)	<0.001	102 (8.4%)	0.003
Cardiovascular disease	2 (0.4%)	199 (1.1%)	0.1	8 (0.7%)	0.6
Chronic kidney disease	2 (0.4%)	202 (1.1%)	0.1	5 (0.4%)	0.9
Smoking	12 (2.6%)	1043 (5.8%)	0.005	38 (3.1%)	0.6
Another comorbidity	32 (7.1%)	655 (3.6%)	<0.001	86 (7.1%)	<0.001

DM – diabetes mellitus, COPD – chronic obstructive pulmonary disease, IQR – interquartile range

*Mann-Whitney U test for continuous variables, χ^2^ test for categorical variables comparing pregnant with positive test vs not pregnant with positive test.

†Mann-Whitney U test for continuous variables, χ2 test for categorical variables comparing pregnant with positive test vs pregnant with negative test.

In the bivariate analysis, hospitalized patients were older than those who received outpatient treatment (41 years, IQR = 33-46 vs 36 years, IQR = 29-42; *P* < 0.001). This was also seen with the control group, in which the significant difference in age was maintained between those who received outpatient treatment (36 years, IQR = 30-42) and those who were hospitalized (41 years, IQR = 34-46; *P* < 0.001).

However, no significant differences in age were found between outpatients and hospitalized patients in the pregnant group. On the other hand, pregnant patients had a higher risk of being hospitalized (RR = 2.10, 95% CI = 1.82-2.55). However, significant differences in the frequency of comorbidities were not found between ambulatory and hospitalized patients (**Table 2**).

**Table 2:** Clinical characteristics of pregnant women according to type of patient care

	No. hospitalized (%) (n = 137)	No. ambulatory (%) (n = 311)	*P* value*
Age, years (median, IQR)	29 (23.5-32.5)	29 (22-33)	0.6
DM	11 (8%)	6 (1.9%)	0.002
COPD	0 (0%)	1 (0.3%)	0.5
Asthma	3 (2.2%)	8 (2.6%)	0.8
Immunosuppression	3 (2.2%)	4 (1.3%)	0.4
Hypertension	5 (3.6%)	12 (3.9%)	0.9
Obesity	22 (16.1%)	37 (11.9%)	0.3
Cardiovascular disease	0 (0%)	2 (0.6%)	0.4
Chronic kidney disease	0 (0%)	2 (0.6%)	0.3
Smoking	3 (2.2%)	9 (2.9%)	0.6
Other comorbidity	13 (9.5%)	19 (6.1%)	0.2

DM – diabetes mellitus, COPD – chronic obstructive pulmonary disease, IQR – interquartile range.

*Mann-Whitney U test for continuous variables, χ2 for categorical variables.

**Figure 2:**
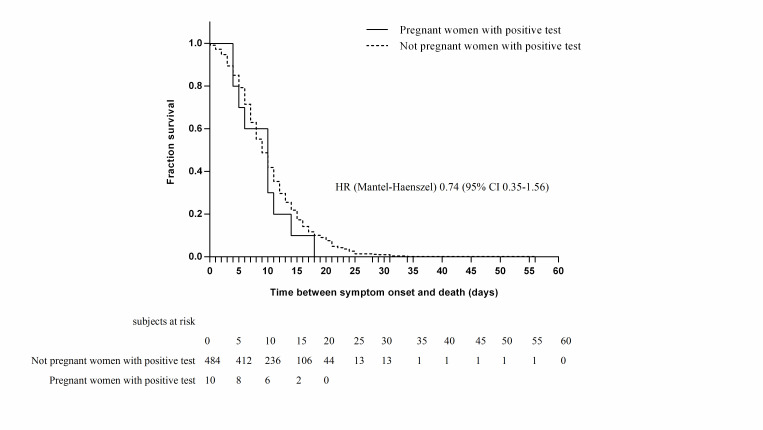
Kaplan-Meier cumulative estimates of propability of death in positive pregnant group compared with positive not pregnant group.

Ten (2.2%) of the pregnant women died. In both pregnant and non-pregnant patients, age was significantly higher among patients who died; the median age of pregnant patients who died was 34.5 years (IQR = 25.5-39) vs 29 years (IQR = 25-32; *P* < 0.001). Nine of the pregnant patients who died had pneumonia, nine were hospitalized, three were in the ICU, and two were managed with mechanical ventilatory assistance; they did not have a higher risk of complications (admission to the ICU, pneumonia, or requirement for mechanical ventilation) or death than the controls in both the bivariate analysis (RR = 0.82, 95% CI = 0.44-1.53) (**Table 3**) and the survival analysis (hazard ratio (HR) = 0.74, 95% CI = 0.35-1.56) (**Figure 2**).

**Table 3:** Clinical evolution of suspected COVID-19 in women of childbearing age

	No. pregnant positive test (%) (n = 448)	No. not pregnant (%) (n = 17942)	*P* value*	No. pregnant negative test (%) (n = 1216)	*P* value†
**Type of patient care**					
Ambulatory	311 (69.4%)	14891 (83%)	<0.001	883 (72.6%)	0.1
Hospitalized	137 (30.6%)	3001 (17%)		333 (27.1%)	
**Pneumonia**	62 (13.8%)	1834 (13.4%)	0.7	74 (6.0%)	<0.001
**Required mechanic ventilation**	7 (5.1%)	174 (5.7%)	0.7	6 (1.8%)	0.04
**Admitted to ICU**	14 (10.2%)	227 (7.4%)	0.2	20 (6.0%)	0.1
**Died**	10 (2.2%)	484 (2.7%)	0.5	7 (0.6%)	0.003
**Days from:**					
Symptoms to admission (median, IQR)	3 (2-5)	4 (2-6)	0.7	2 (1-4)	<0.001
Admission to death (median, IQR)	7.5 (2.75-9.75)	5 (2-9)	0.5	1 (0-2)	0.07
Symptoms to death (median, IQR)	10 (4.75-11.75)	9 (6-14)	0.9	4 (2-6)	0.03

ICU – Intensive care unit, IQR – interquartile range

*Mann-Whitney U test for continuous variables, χ2 test for categorical variables comparing pregnant with positive test vs not pregnant with positive test.

†Mann-Whitney U test for continuous variables, χ2 test for categorical variables comparing pregnant with positive test vs pregnant with negative test.

In the multivariate analysis, each comorbidity was entered into the model with age and pregnancy status, but only the history of DM and chronic kidney disease remained independently associated with death for SARS-CoV-2 in the positive cohort (**Table 4**).

**Table 4:** Bivariable and multivariable analysis of potential prognostic variables associated with 60-d COVID-19 mortality in women of childbearing age

	Univariable (RR, 95%)	Multivariable (HR, 95% CI)*
**DM:**		
no	1 (ref)	1 (ref)
yes	5.09 (4.47-5.78)	1.23 (1.02-1.49)
**COPD:**		
no	1 (ref)	1 (ref)
yes	6.41 (3.49-11.77)	1.33 (0.75-2.36)
**Asthma:**		
no	1 (ref)	1 (ref)
yes	1.08 (0.72-1.62)	0.79 (0.51-1.26)
**Immunosuppression:**		
no	1 (ref)	1 (ref)
yes	3.95 (2.59-6.01)	1.48 (0.97-2.26)
**Hypertension:**		
no	1 (ref)	1 (ref)
yes	3.66 (3.19-4.22)	1.05 (0.94-1.40)
**Obesity:**		
no	1 (ref)	1 (ref)
yes	1.79 (1.58-2.03)	1.01 (0.84-1.22)
**Chronic kidney disease**		
no	1 (ref)	1 (ref)
yes	9.44 (6.80-13.01)	1.72 (1.25-2.38)
**Cardiovascular disease:**		
no	1 (ref)	1 (ref)
yes	2.31 (1.30-4.12)	0.84 (0.34-2.03)
**Smoking:**		
no	1 (ref)	1 (ref)
yes	0.81 (0.542-1.21)	1.14 (0.75-1.73)
**Other comorbidity:**		
no	1 (ref)	1 (ref)
yes	1.83(1.31-2.57)	0.85 (0.58-1.25)

RR – relative risk, HR – hazard ration, DM – diabetes mellitus, COPD – chronic obstructive pulmonary disease

*Corrected for age and pregnancy status.

### Pregnant women with suspected COVID-19

We compared pregnant patients with suspected COVID-19 and a positive test vs those with a negative test. Bivariate analysis showed that pregnant patients with a positive test were older than patients with a negative test, and they had a higher frequency of obesity (**Table 1**).

**Figure 3:**
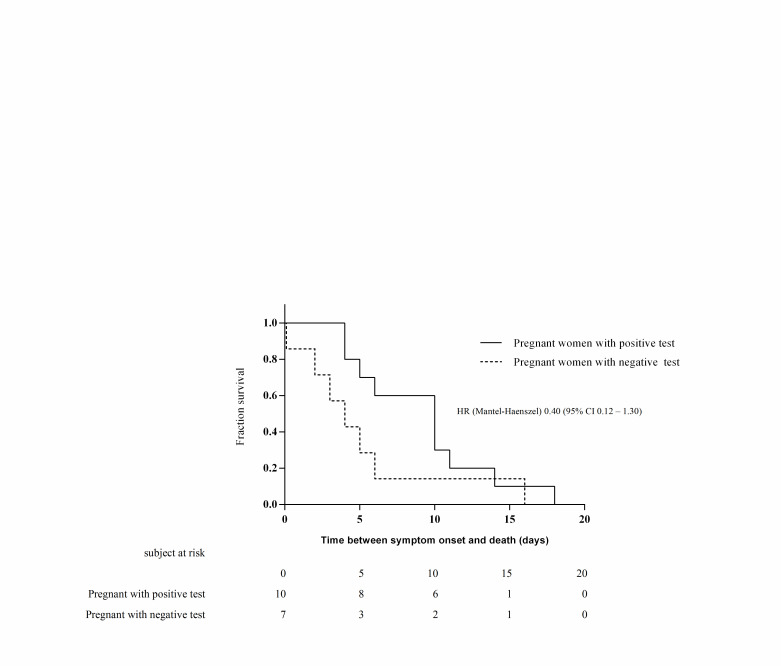
Kaplan-Meier cumulative estimates of propability of death in positive pregnant group compared with negative pregnant group.

Regarding clinical evolution, we did not find differences in the type of medical care received by patients with or without a positive test. Seven (0.6%) pregnant women with a negative test died; these patients had fewer days from the beginning of symptoms until admission or death and from admission to death with respect to pregnant women with a positive test (Table 3). Three of these patients were diagnosed with pneumonia, two were admitted to the ICU, and one required mechanical ventilation. In bivariate analysis, patients with a positive test had a higher risk of death than patients with a negative test (RR = 3.87, 95% CI = 1.48-10.12), but we found no significant difference in 60-day mortality (HR = 0.40, 95% CI = 0.12-1.30) (**Figure 3**). In the multivariate analysis, obesity remained a risk factor for the risk of a positive test in pregnant patients (**Table 5**).

**Table 5:** Bivariable and multivariable analysis of potential prognostic variables associated with a SARS-COV-2 positive test in pregnant women with suspected COVID-19

	Univariable (RR, 95%)	Multivariable (HR, 95% CI)*
**DM:**		
no	1 (ref)	1 (ref)
yes	0.59 (0.88-2.86)	1.07 (0.64-1.78)
**COPD:**		
no	1 (ref)	1 (ref)
yes	0.90 (0.09-8.66)	1.37 (0.17-10.93)
**Asthma:**		
no	1 (ref)	1 (ref)
yes	0.50 (0.26-0.95)	0.46 (0.25-0.86)
**Immunosuppression:**		
no	1 (ref)	1 (ref)
yes	0.79 (0.34-1.82)	1.20 (0.55-2.60)
**Hypertension:**		
no	1 (ref)	1 (ref)
yes	1.2 (0.69-2.12)	1.41 (0.85-2.36)
**Obesity:**		
no	1 (ref)	1 (ref)
yes	1.56 (1.16-2.12)	1.36 (1.02-1.81)
**Chronic kidney disease:**		
no	1 (ref)	1 (ref)
yes	1.08 (0.21-5.57)	2.01 (0.40-8.49)
**Cardiovascular disease:**		
no	1 (ref)	1 (ref)
yes	0.67 (0.14-3.18)	0.59 (0.66-1.37)
**Smoking:**		
no	1 (ref)	1 (ref)
yes	0.85 (0.45-1.62)	0.78 (0.44-1.40)
**Another comorbidity:**		
no	1 (ref)	1 (ref)
yes	1.01 (0.68-1.49)	0.95 (0.66-1.37)

RR – relative risk, HR – hazard ratio, DM – diabetes mellitus, COPD – chronic obstructive pulmonary disease

*Corrected for all variables and age.

## DISCUSSION

The analysis presented here represents the largest cohort of pregnant women laboratory-tested for COVID-19 from a single country. In our results, we found a lethality of 2.2% among pregnant women. We also found that pregnant women positive for COVID-19 did not have an increased risk of mortality or complications with respect to a control group of women of childbearing age who were not pregnant but positive for COVID-19. Similarly, pregnant women with confirmed COVID-19 had no higher risk of death than pregnant women with a negative test.

Most previous publications have been case series with a very low number of patients [13], and some systematic reviews were carried out analyzing these case series. A systematic review of 108 pregnant patients included mostly women from China, with some cases from Sweden, the USA, Korea, and Honduras, with ages similar to our cohort. In this review, 3% of patients were admitted to the ICU and no deaths were reported [14]. In another systematic review, Elshafeey et al. [15] analyzed 33 studies including 385 pregnant women; they reported one maternal death and lower frequencies of admission to the ICU or requirement for mechanical ventilation than our cohort. However, they did not make a comparison with a control group and the risk of complications could not be estimated. Another case series [16] compared the evolution of pregnant patients with COVID-19 and their positive contacts; they observed a more torpid evolution in pregnant women than their contacts but could not reach conclusions due to a low case number.

We identified that pregnant patients with a positive test have a higher risk of being hospitalized than non-pregnant patients with a positive test, which could be due to, in addition to the respiratory symptoms associated with COVID-19, they had obstetrics indications for hospitalization as indicated by the Mexican guidelines for the care of pregnancy, childbirth, and the puerperium during the COVID-19 pandemic [17]. Unfortunately, the obstetric characteristics of the patients are not specified in the database.

The presence of chronic degenerative diseases is related to an increased risk of complications and death from COVID-19 [18], including in Mexico [19]. However, as other authors indicated previously [20], the impact of these highly prevalent diseases on the clinical evolution of COVID-19 in pregnant women has not been analyzed. We found that DM and chronic kidney disease are risk factors for death among patients of childbearing age with a positive test, but were not found as risk factors for mortality among pregnant patients with or without a positive test. However, obesity was a risk factor for a positive test in pregnant women with suspected COVID-19.

Moreover, we identified that the patients with a negative test had a shorter duration from the onset of symptoms until death than pregnant women with positive test, which may be because the causes of death in these patients were not related to respiratory symptoms, which were classified as suspicious. Unfortunately, the causes of the deaths were not registered, yet the pregnant patients with a positive test had a higher risk of death than the patients with a negative test. Therefore, it seems that pregnant patients with a negative test die less frequently but more quickly than pregnant patients with a positive test. In addition, only data from pregnant women suspected of COVID-19 are included in this study, and could not be compared to other causes of mortality in pregnant women with negative tests and without respiratory disease. Because of this, we consider it more appropriate to compare the risk of pregnant patients with a positive test and non-pregnant patients with a positive test, which showed that pregnancy does not increase the risk of death.

This study has several limitations. First, information on the obstetric characteristics of the pregnant patients, such as gestational age, resolution of pregnancy, obstetric diseases and complications, and perinatal history, was missing. In addition, the symptoms presented by the patients were unknown. Other causes of hospitalization, ICU admission, and death could not be analyzed because the database only includes women suspected of having COVID-19. Therefore, the role of COVID-19 in the general mortality of pregnant women was not established.

On the other hand, this study is a large cohort from a single country to analyze the risk of complications and mortality in pregnant patients with RT-PCR-confirmed COVID-19, and we performed a comparison with two control groups: COVID-19-positive women of childbearing age and pregnant women with respiratory symptoms but a negative test for SARS-CoV-2. We also estimate the association between the presence of complications and death and the history of pre-existing comorbidities, mainly chronic-degenerative diseases. We found that pregnancy is not a risk factor for COVID-19 mortality, but pregnant women had a higher risk of being hospitalized and a higher frequency of ICU admission than non-pregnant women, and they had a lower median age and reduced non-specified comorbidities. In Cox regression analysis, diabetes and chronic kidney disease remain risk factors for mortality in the cohort.

Since the beginning of the pandemic, pregnant patients have been identified as a vulnerable group for complications and risk of death from COVID-19 due to previous experiences, such as pandemic influenza. This is likely why medical staff decide more frequently on the hospitalization when pregnant patients ask for medical attention, and they may be more careful in identifying COVID-19 complications earlier. These results offer solid evidence of SARS-CoV-2 infection, how severe it is, and outcomes in the specific context of pregnancy. We provide evidence supporting specific guidelines for pregnancy care during SARS-COV-2 infection.

## CONCLUSIONS

In Mexico, pregnant patients with laboratory-confirmed COVID-19 do not have a greater risk of complications or death from COVID-19 than non-pregnant patients with laboratory-confirmed COVID-19 or pregnant patients with a negative test. However, the presence of DM and chronic kidney disease increases the risk of death in women of childbearing age, but not particularly in pregnant patients. The causes of death in pregnant patients with symptoms suspicious of COVID-19 but a negative test need to examined more thoroughly, as these patients present with a faster evolution toward death.
